# A case report of Chinese brothers with inherited *MECP2-*containing duplication: autism and intellectual disability, but not seizures or respiratory infections

**DOI:** 10.1186/1471-2350-13-75

**Published:** 2012-08-21

**Authors:** Xiu Xu, Qiong Xu, Ying Zhang, Xiaodi Zhang, Tianlin Cheng, Bingbing Wu, Yanhua Ding, Ping Lu, Jingjing Zheng, Min Zhang, Zilong Qiu, Xiang Yu

**Affiliations:** 1 Department of Child Healthcare, Children’s Hospital of Fudan University, Shanghai, China; 2 Institute of Neuroscience and State Key Laboratory of Neuroscience, Shanghai Institutes for Biological Sciences, Chinese Academy of Sciences, Shanghai, China; 3 Graduate School of the Chinese Academy of Sciences, Shanghai, China

**Keywords:** MECP2, Autism, ASD, CNV, Chinese patients

## Abstract

**Background:**

Autistic spectrum disorders (ASDs) are a family of neurodevelopmental disorders with strong genetic components. Recent studies have shown that copy number variations in dosage sensitive genes can contribute significantly to these disorders. One such gene is the transcription factor *MECP2*, whose loss of function in females results in Rett syndrome, while its duplication in males results in developmental delay and autism.

**Case presentation:**

Here, we identified a Chinese family with two brothers both inheriting a 2.2 Mb *MECP2*-containing duplication (151,369,305 – 153,589,577) from their mother. In addition, both brothers also had a 213.7 kb duplication on Chromosome 2, inherited from their father. The older brother also carried a 48.4 kb duplication on Chromosome 2 inherited from the mother, and a 8.2 kb deletion at 11q13.5 inherited from the father. Based on the published literature, *MECP2* is the most autism-associated gene among the identified CNVs. Consistently, the boys displayed clinical features in common with other patients carrying *MECP2* duplications, including intellectual disability, autism, lack of speech, slight hypotonia and unsteadiness of movement. They also had slight dysmorphic features including a depressed nose bridge, large ears and midface hypoplasia. Interestingly, they did not exhibit other clinical features commonly observed in American-European patients with *MECP2* duplication, including recurrent respiratory infections and epilepsy.

**Conclusions:**

To our knowledge, this is the first identification and characterization of Chinese Han patients with *MECP2-*containing duplications. Further cases are required to determine if the above described clinical differences are due to individual variations or related to the genetic background of the patients.

## Background

Autism spectrum disorders (ASDs) are neurodevelopmental disorders with complex etiology and strong genetic basis, characterized by impaired communication, reduced social interaction, and stereotyped and/or repetitive behavior [1-4]. Over the past decade, an emergent feature regarding the genetics of ASD is the importance of gene dosage, where both loss and gain of function of a gene can result in autistic phenotypes [4,5]. A prominent example is the Methyl-CpG-binding Protein 2 gene (*MECP2*; MIM: 300005), located at Xq28. Loss of function of one copy of *MECP2* leads to Rett syndrome (RTT; MIM 312750), a progressive neurodevelopmental disorder characterized by loss of motor skills and communication abilities, as well as stereotypic hand movements and other autistic features, occurring in 1:10,000 girls [6-9]. More recently, duplication of the *MECP2* gene has been found in boys with developmental delay, intellectual disability and/or autism in a series of studies [10-28]. Core features of the syndrome included infantile hypotonia, mild dysmorphic features, developmental delay, intellectual disability, abnormal movement and absent to minimal speech. Although not all studies examined autistic characteristics, when the examinations were carried out, autistic phenotypes were prominent among patients with *MECP2* duplication [15,21,23,29]. In fact, of 8 boys evaluated using the Autism Diagnostic Observation Schedule (ADOS) in one study, 7 (88%) exceeded the cutoff score for autism, while the remaining one exceeded the score for ASD [23]. Thus altering the gene dosage of *MECP2* by both deletion and duplication can generate autistic phenotypes. Corroborating these clinical findings, similarities in phenotypes, including autistic features, were observed both in mouse models of *MECP2* deletions and duplications [30-34].

Whether *MECP2* duplication is a cause of intellectual disability and/or autism in the Chinese Han population is unknown. A previous study screening 82 Chinese Han boys diagnosed with autism using real-time quantitative polymerase chain reaction (qPCR) failed to identify deletions or duplications in *MECP2*[35]. Since the sample size was small, the question of whether *MECP2* duplication is present in Chinese patients diagnosed with autism required further study. Here, we report two brothers diagnosed with autistic disorder carrying duplication in *MECP2*, inherited from their mother. Detailed examination, medical history inquiry and characterization by ADOS showed that these boys shared many characteristics with previously reported patients carrying duplication encompassing the *MECP2* gene [10-28], including autism, intellectual disability, hypotonia and mild dysmorphic features, but not recurrent respiratory infections or epilepsy. Genome-wide CNV scan using Agilent 1 M comparative genomic hybridization (CGH) microarray showed that both brothers carried a 2.22 Mb *MECP2-*duplication containing CNV, inherited from their mother. To our knowledge, this is the first report and characterization of *MECP2* duplication patients from the Chinese Han population.

## Case presentation

**Figure 1:**
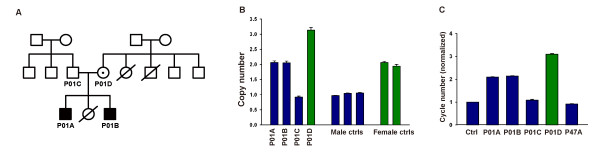
**Identification of two brothers with inherited *MECP2 *duplication in Chinese autism patients. A**. Pedigree of family P01, standard pedigree symbols are used. **B**. CNV screening using Accucopy kit for all members of family P01. **C**. Confirmation of results using real-time qPCR. P47A is a boy with normal *MECP2* gene dosage. Males are represented by blue bars, and females by green bars. Error bars represent s.e.m.

In a screen for CNVs in *MECP2* carried out for children diagnosed with ASD at the Children’s Hospital of Fudan University, 53 unrelated male subjects diagnosed with ASD using *DSM-IV* (average age at first diagnosis: 4.15 ± 0.27 years) were tested. One boy (P01A) was identified with a *MECP2* duplication. This patient also had an older brother with intellectual disability and no speech. The patient, his older brother and both parents were then all assayed for CNVs in *MECP2* using an Accucopy kit, a multiplex competitive amplification-based method (Genesky Biotechnologies Inc., Shanghai, China, see Additional file 1 for details) [36]. The older brother (P01B) was found to have two copies of *MECP2*, the father (P01C) had a single copy, while the mother (P01D) carried three copies (Figure 1A, B). Control males and females carried one or two copies of *MECP2*, respectively, as expected (Figure 1B). These results, further confirmed using standard real-time qPCR (Figure 1C), showed that both brothers carried *MECP2* duplication inherited from the mother.

### Clinical summary

Detailed clinical data was gathered from the family. The pedigree is shown in Figure 1A. A girl (G2 P2), born between the two brothers, was born prematurely at 30 weeks, likely because the mother was startled. She died one month after birth. It was noted that she fed very poorly, although her body weight did increase slightly. On the day before she died, she did not feed at all. The cause of death was unknown, as no medical examinations were carried out. It is possible that she also carried the *MECP2*-containing duplication (no DNA sample available). Although DNA from other members of the mother’s family could not be obtained, the pedigree showed that the mother was the youngest of 5 children, with 2 healthy brothers, and one brother and one sister who died at a young age. The healthy brothers already have grandchildren, and both they and their progeny have no significant health or psychological issues. The older sister died before P01D was born, likely from an accident. The other brother died shortly after birth, for unknown causes. The mother of P01D is over eighty years old, of good health, apart from high blood pressure, and has normal intellectual and social abilities.

#### Patient 01A

Patient 01A (G3 P3 L2) was born at full-term to healthy, nonconsanguineous parents following an uneventful birth, at a birth weight of 3400 g. He showed general developmental delay and was able to walk at the age of 20 months. When examined at the age of 9 years and 5 months, he walked and ran slightly unsteadily and could not jump with both feet off the ground. He was in the normal height range when compared to children of his age, but was slightly slender. In terms of facial features, he had large ears and a depressed nasal bridge. He was slightly hypotonic, but had no history of respiratory infections, has not undergone operations or major medical treatments and has had no seizures or epilepsy. He had no sleep disturbances, but was often constipated. He occasionally got a cold, but was not ill very often. He was hospitalized once at the age of a few months. At the age of 3–4 years, he generated a couple of words, including “mama” and “tea”. Currently, he could only say “mama” and had passive understanding of simple sentences as spoken by his mother, but no other language or echolalia. He could eat a pear by himself, but needed to be fed by his mother for meals, due to difficulty holding a spoon. He occasionally ran around with other children, but could not engage in interactive play. He got very anxious when encountering strangers or when entering a new environment. When happy, he clapped his hands.

Cognitive/developmental evaluation using the Bayley Scales of Infant Development-3 [37] showed that he had cognitive and language skills, as well as motor skills well below that for his age (Table 1). This patient, who was diagnosed with autism using DSM-IV [38] at 4 years and 11 months, had an ADOS score [39] (Table 1) of 20 at the time of this examination (9 years and 5 months), well above the cutoff for autistic disorder.

**Table 1:** **Quantification of developmental, cognitive and autism phenotypes of *MECP2 *duplication-containing patients**

**Subject**	**P01A**	**P01B**
Age at examination	9 years 5 months	18 years
**Bayley Scales of Infant Development-3**		
Developmental age	9 months	6 months
Receptive language	15 months	11 months
Expressive language	6 months	5 months
Fine motor	15 months	12 months
Gross motor	15 months	15 months
**Weschler Intelligence Scale**		
Verbal IQ	< 40	< 36
Performance IQ	< 40	< 14
Full Scale IQ	< 40	< 24
**Autism Diagnostic Observation Schedule (ADOS) scores**		
ADOS communication score	6	5
(cutoff score for autism is ≥4)		
ADOS social interaction score	14	12
(cutoff score for autism is ≥7)		
Combined ADOS score (cutoff score for autism is ≥12)	20	17

#### Patient 01B

Patient 01B (G1 P1), the older brother of patient 01A, was 18 years old at the time of examination. He was of normal height and weight when compared to his peers. He had mild dysmorphic features including a depressed nasal bridge and midface hypoplasia. He had no history of respiratory infections or other major medical treatments and no history of seizures or epilepsy. He has not been hospitalized, and is otherwise healthy apart from an occasional cold. A previous computed tomography (CT) scan showed no obvious abnormalities. He learnt to walk at 20 months and currently walked and ran slightly unsteadily. He liked to walk on his toes, but could not jump with both feet off the floor. He had no language, but could passively understand simple phrases as spoken by his mother. He was able to eat by himself, and stayed in his room alone at all times other than meal times. He liked to look at cars on the street and was very excited by wheels. When happy, he put his hands to his mouth and laughed loudly. He flapped his hands a lot and did not engage in interaction with others, but laughed often. Although this patient was 18 years at the time of examination, his cognitive, language and motor skills were in the range of 6 to 15 months as measured by Bayley Scales of Infant Development-3 [37] (Table 1). His ADOS score of 17 [39] was well above the cutoff for autistic disorder (Table 1).

#### Parents of the patients P01A and P01B

Since previous publications have shown that female carriers of *MECP2* duplications had psychiatric symptoms, the mother of the patients (P01D) completed a Symptom Checklist-90 [40,41], and both parents completed the Wechsler Adult Intelligence Scale-Revised [42,43] and the Broad Autism Phenotype Questionnaire (BAPQ) [44]. Both parents scored in the normal range on the Wechsler Intelligence Scale (Table 2). In terms of the broad autism phenotype, both parents scored slightly higher than average, with the mother scoring further away from the cutoff score than the father (Table 2). Consistent with a previous publication [23], the mother of the patients, who also carried the *MECP2-*containing duplication, had slightly higher scores in somatization, depression, anxiety and psychoticism on completing Symptom Checklist-90 [40,41] and reported sleep disturbances. She reported no other healthy issues, and has not had any miscarriages.

**Table 2:** C**linical summary of the parents of *MECP2 *duplication-containing patients**

**Subject**	**Father (P01C)**	**Mother (P01D)**
**Wechsler Adult Intelligence Scale-Revised**		
Verbal IQ	93	92
Performance IQ	138	95
Full Scale IQ	109	93
**Broad Autism Phenotype Questionnaire (cutoff score in brackets)**		
Aloof Personality (≤3.25)	3.33	3.92
Pragmatic language deficits (≤2.75)	3.00	2.83
Rigid personality (≤3.50)	3.50	3.75
Total score (≤ 3.15)	3.28	3.50
**Symptom Checklist-90**		
		Slightly high scores in somatization, depression, anxiety, psychoticism; normal in all other categories

### Determination of the precise duplication interval by aCGH

**Figure 2:**
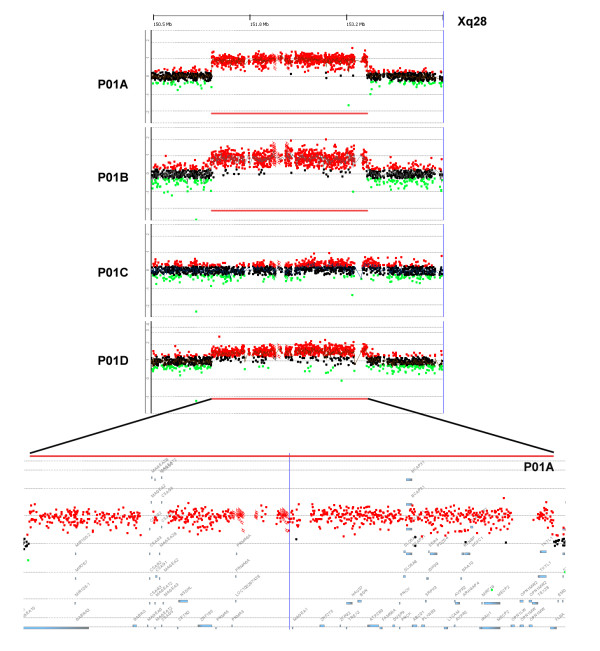
**Identification of a 2.22 Mb *MECP2 *duplication in P01A and P01B, inherited from their mother. **Dots represent relative intensities in log2 ratio and genomic locations of the oligonucleotide probes employed in aCGH assay. For males (P01A, P01B, P01C), green (loss), black (no change), and red (gain) dots represent log2 ratio deviation from the horizontal line of 0; and for the female (P01D), green (loss), black (no change), and red (gain) dots represent log2 ratio deviation from the horizontal line of 1. Regions with copy number gains are indicated with red horizontal bars.

To determine the precise size of the *MECP2* duplication and to assay if additional CNVs are present in this family, Agilent SurePrint G3 Human CGH Microarray 1x1M was used to assay the entire genome of all four members of the family (performed by Shanghai Biotechnology Corporation, Shanghai, China; see Additional file 1 for details). The *MECP2* containing duplication (151,369,305 – 153,589,577) was found to be 2.22 Mb, including a large number of genes other than *MECP2* (Figure 2). The duplicated region, starting with *GABRA3* and ending with *FLNA*, is the same in the brothers and their mother, consistent with both brothers inheriting the CNV from their mother.

Both Patients 01A and 01B also had a 213.7 kb duplication on Chromosome 2, inherited from their father. This region contained the genes *PFN4*, *LOC375190*, *C2orf84* and *ITSN2*. None of these genes have been shown to be associated with autism, and furthermore, since the father is a healthy, normal adult, this CNV is unlikely to be disease-causing by itself. Patient 01A had no other CNVs. Patient 01B had a 48.4 kb duplication on Chromosome 2, containing no known genes, inherited from the mother, and a small deletion (8.2 kb) at 11q13.5, containing *GDPD4*, inherited from his father. In a previous report [45], loss of *GDPD4* was found as a low frequency *de novo* CNV in autism patients (3 autism from 1683 analyzed) but not in normal controls. The relationship of *GDPD4* to autism is otherwise unknown. In our study, since the *GDPD4*-containing CNV in Patient 01B is inherited from a healthy parent, it is unlikely to be disease-causing by itself.

Since, based on the existing literature, the other CNVs found in Patients P01A and P01B are unlikely to be the main cause of the patients’ phenotypes, our whole genome CNV results provided further evidence that the main genetic abnormality in patients P01A and P01B is their *MECP2*-containing duplication, inherited from the mother. The other CNVs could also, in principle, contribute to the patients’ phenotypes in the background of the *MECP2-*containing duplication. As the *MECP2-*containing duplication region is relatively large, we also examined whether other genes in the duplicated region could contribute to the phenotype. As shown in Figure 2 and Additional file 2, this region contained 54 genes, including two subunits of the GABA receptor (*GABRA3*, *GABRQ*), an isocitrate dehydrogenase (*IDH3G*), interleukin-1 receptor-associated kinase 1 (*IRAK1*), the L1 cell adhesion molecule (*L1CAM*), a PDZ domain protein (*PDZD4*), Plexin B3 (*PLXNB3*) and a creatine transporter (*SLC6A8*) (More details below). Estimation of the association level of this group of genes with autism was carried out using the AutismKB database (http://autismkb.cbi.pku.edu.cn/index.php), an online database that assigned a weighted score to each gene, CNV or linkage region reported to be associated with autism, based on published literature [46]. Using this database, we found that although *MECP2* was by far the gene most associated with autism, with a high score of 26, many other genes in the interval also contributed from 3 to 12 points, bringing the score of the entire duplicated region to a very high total of 158 (Additional file 2). The result of this analysis further underscored the importance of this *MECP2*-containing region to autism.

### Size of duplicated region

Compared to other patients with reported duplications in *MECP2*, the duplicated region of 2.2 Mb identified in family P01 is relatively large. The duplication size in the reported literature is from 0.32 Mb [23] to 2.56 Mb [47]. The centromeric end of the duplication is located in the 3’ untranslated region of *GABRA3* (located on the complementary strand), suggesting potential duplication of a functional copy of the *GABRA3* gene. The telomeric end is within *FLNA*. Since this gene is also located on the complementary strand, and thus the 5’ end and promoter regions are outside of the duplicated region, it is likely that there is no gain-of-function of *FLNA*. Consistently, the patients did not exhibit the severe, chronic intestinal peudo-obstruction phenotype associated with *FLNA* duplication [14]. Also worth noting, the telomeric breakpoint is within the 215 kb region distal to *MECP2* that has been previously identified as a genomic interval prone to rearrangements resulting in *MECP2* duplications [47].

Although a number of genes in the duplication region have been reported to be associated with autism, the clinical characteristics of our patients fit well with patients carrying just *MECP2* duplication [15,20,23,47], the most autism-related gene within the region. *IRAK1*, the gene neighboring *MECP2* and also included in the minimum duplication region [15,20,23,47], is a member of the toll-like receptor-signaling pathway [48] and has been proposed to contribute to the recurrent infection phenotype in affected boys [26]. Interestingly, although P01A and P01B had Xq28 duplications that clearly included *IRAK1*, they did not have the recurrent infections reported in some other patients.

Another gene in the duplicated interval with developmental phenotypes is *SLC6A8*, a creatine transporter whose loss of function resulted in severe intellectual disability and autism [49,50]. In other *MECP2* duplication patients, where the duplicated region also included *SLC6A8*, creatine levels in spinal fluid and urine were normal, and there were no additional phenotypes [20]. Similarly, our patients did not report any metabolic deficiencies.

The other genes in the duplicated interval reported to be associated with autism are mostly based on CNV studies, rare mutations found during X-chromosome sequencing and/or expression studies (see scores in Additional file 2 and references within the AutismKB database). Since there are no detailed patient descriptions in these large scale studies, it is difficult to establish genotype-phenotype relationships for these genes. It is possible that some of these genes may affect the patient’s clinical characteristics in the background of *MECP2* duplication. Of particular interest in this group are GABA receptor subunits *GABRA3* and *GABRQ*, components of GABAergic neurotransmission. GABA is the main inhibitory neurotransmitter in the brain, and alterations of GABAergic signaling are thought to be associated with ASD [51]. Also of interest are synaptic components and cell adhesion molecules, which have recently been associated with autism [52]. Duplicated genes in this category include: *L1CAM*, encoding the L1 cell adhesion molecule; *PDZD4*, the PDZ domain containing and potential post-synaptic scaffold protein, and *PLXNB3*, the axon guidance molecule Plexin B3, which acts as a receptor for the Semaphorin 5A signal.

### Clinical features of boys with duplicated *MECP2*

Our results are consistent with previous reports showing lack of correlation between duplication size and clinical phenotypes, as the patients we identified are no more severe than those with much smaller duplications [14,15,23,26,27,47].

## Conclusions

In this study, we identified a Chinese Han family with two brothers carrying a *MECP2*-containing duplication inherited from their mother. The duplicated region, as defined by microarray analysis, was 2.22 Mb in size. To our knowledge, this is the first report of Chinese Han autistic patients with *MECP2* duplication. These individuals shared many characteristics with previously reported *MECP2* duplication patients [9-27], including autism, intellectual disability, hypotonia and mild dysmorphic features, but not recurrent respiratory infections or epilepsy. Identification and characterization of more patients are needed for studies regarding potential differences in phenotype between ethnic groups, as well as for the correlation between duplication size and clinical phenotype. Importantly, our results demonstrate that *MECP2* duplication is present in autism patients of Chinese Han ethnicity, likely at an occurrence rate suitable for genetic testing.

## Consent

This study was approved by the Ethics Committee of the Children’s Hospital of Fudan University (Approval number: Children’s Hospital of Fudan University Ethics Protocol 2011–040; Title: Research on the behavioral phenotype and genetic basis of autism spectrum disorder). Written informed consent for the collection of peripheral blood samples and subsequent analyses was obtained from all participating families, with the parents giving consent for themselves and on behalf of their minor children. A copy of the written consent is available for review by the Editor-in-Chief of this Journal.

## Competing interests

The authors declare no competing financial interests.

## Author contributions

XX, ZQ and XY conceived the study and participated in its design and coordination; XX, QX, YZ, BW, YD, and PL identified the patients and carried out the clinical characterizations; XZ, TC, JZ, and MZ carried out the molecular genetics studies; XY wrote the manuscript; all authors read and approved the final manuscript.

## Pre-publication history

The pre-publication history for this paper can be accessed here:

http://www.biomedcentral.com/1471-2350/13/75/prepub

## Supplementary Material

Additional file 1 **Supplemental Methods Measuring*MECP2-*containing CNVs Using AccuCopy Kit. Real-time qPCR. Agilent 1M CGH and data analysis.** Click here for file

Additional file 2 Genes in Xq28 duplicated in P01A and P01B, listed together with their AutismKB scores. Click here for file
