# Supplementary material for: A case report of Chinese brothers with inherited MECP2-containing duplication: autism and intellectual disability, but not seizures or respiratory infections
Source: BMC Med Genet. 2012 Aug 21;13:75. doi: 10.1186/1471-2350-13-75 (PMC3506511; doi:10.1186/1471-2350-13-75)
Supplement: Additional file 1 — Supplemental Methods MeasuringMECP2-containing CNVs Using AccuCopy Kit. Real-time qPCR. Agilent 1M CGH and data analysis. [file 1471-2350-13-75-S1.doc]

**Additional file 1 - Supplemental methods**

Measuring *MECP2-*containing CNVs Using AccuCopy Kit

Three sets of primers targeting *MECP2* were designed using Primer3 software (http://frodo.wi.mit.edu/). The copy number of these three *MECP2* target DNA fragments was measured using a custom-designed Multiplex AccuCopy Kit (Cat#:CN0105, Genesky Biotechnologies Inc., Shanghai, China) . The *MECP2* primers amplified the following fragments on the X Chromosome: 153,357,667 - 153,357,746, 153,295,798 - 153,295,884, and 153,297,825 - 153,297,975. Nucleotide positions are as in the human genome GRCh37/hg19 assembly.

Each reaction was carried out by mixing the synthesized competitive double stranded DNA for target and reference genes together with a defined amount of sample DNA. A multiplex competitive PCR was then performed to simultaneously amplify all reference and target genes from both sample and competitive DNA using multiple fluorescence-labeled primer pairs. Briefly, the 20 μl PCR reaction for each sample contained 1x AccuCopy PCR Master Mix, 1x Fluorescence Primer Mix, 1x Competitive DNA mix and ~10 ng sample DNA. The PCR program used was: 95℃ 10 min; 11 cycles of 94℃ 20 s, 65℃-0.5℃/cycle 40 s, 72℃ 1.5 min; 24 cycles of 94℃ 20 s, 59℃ 30 s, 72℃ 1.5 min; 60℃ 60 min. PCR products were diluted 20-fold before loading onto ABI3730XL sequencer (Applied Biosystems, USA) to separate amplicons of different size by capillary electrophoresis. Raw data was analyzed using GeneMapper4.0, and the peak ratio of sample DNA to competitive DNA (S/C ratio) for all target and reference fragments were exported to Excel.

The S/C ratio of each *MECP2* target fragment were first normalized to the S/C ratio of reference genes (RPP14, TBX15), and then further normalized to the median of the group data. The median was used for the normalization, rather than the mean, so that those patients with CNVs would not significantly affect the group data. As *MECP2* is located on the X chromosome, and most of our tested subjects are male (apart from the mother of one patient and control females), the median was taken from data of male subjects only. The normalized data was first averaged for the two reference genes (RPP14, TBX15) and then averaged between the 3 *MECP2* primer sets. For each patient, error bar represents s.e.m. and “n” is the 3 independent *MECP2* primers.

Real-time qPCR

Patients identified as potentially carrying *MECP2* duplication by AccuCopy were further verified using real-time qPCR. The mix contained 20 ng genomic DNA, SYBR Premix Ex Taq (TaKaRa, Cat# DRR041A), and human *MECP2* primers (forward primer 5'-CCAGGTCATGGTGATCAAAC, reverse primer 5'-TCCTGCACAGATCGGATAG) or human *TBP* primers (forward primer 5'-GCAGGCAACACAGGGAACCT, reverse primer 5'-GAACTCTCCGAAGCTGGCGT), and was carried out on a LightCycler 480 Real-Time PCR System (Roche Applied Science). Triplicates were performed and *MECP2* data was normalized relative to *TBP*.

Agilent 1M CGH and data analysis

Whole genome CNV analysis was carried out for the family identified as having *MECP2* duplication using Agilent SurePrint G3 Human CGH Microarray 1x1M (Agilent, Cat# G4824A-021529). Control samples were obtained from a mix of 31 healthy and normally developing boys from a local kindergarten, aged 3-5. Microarray hybridization, data generation and normalization were performed by Shanghai Biotechnology Corporation (SBC, Shanghai, China) following standard Agilent protocols. Data analysis and CNV identification were performed on Agilent Genomic Workbench Lite Edition 6.5. The genomic copy number was defined by analysis of the normalized log_2_ (Cy5/Cy3) ratio average of the CGH signal, amid a 10 kb window. For analysis of autosomes, the thresholds for duplication and deletion were respectively 0.5 and -0.8; for X Chromosomal analysis of males, the values were 0.8 and -0.8 respectively; for X Chromosomal analysis of the mother, since the controls were all males, a threshold of 1.8 was used for duplication, and 0.2 for deletion. All CNVs were checked against the Database of Genomic Variants (http://projects.tcag.ca/variation/) to exclude those present in healthy individuals. Nucleotide positions are as in human genome GRCh37/hg19 assembly.

**References**

1. Du R, Lu C, Jiang Z, Li S, Ma R, An H, Xu M, An Y, Xia Y, Jin L *et al*: **Efficient typing of copy number variations in a segmental duplication-mediated rearrangement hotspot using multiplex competitive amplification**. *J Hum Genet* 2012.
