# Supplementary material for: A case report of Chinese brothers with inherited MECP2-containing duplication: autism and intellectual disability, but not seizures or respiratory infections
Source: BMC Med Genet. 2012 Aug 21;13:75. doi: 10.1186/1471-2350-13-75 (PMC3506511; doi:10.1186/1471-2350-13-75)
Supplement: Additional file 2 — Genes in Xq28 duplicated in P01A and P01B, listed together with their AutismKB scores. [file 1471-2350-13-75-S2.doc]

**Additional file 2 – Table S1**

**Table S1: Genes in Xq28 duplicated in P01A and P01B, listed together with their AutismKB scores**

| **Gene** | **AutismKB score** |
| --- | --- |
| *ARHGAP4* | 4 |
| *ATP2B3* | 7 |
| *AVPR2* | 3 |
| *BCAP31* | 6 |
| *FLNA* | 3 |
| *GABRA3* | 6 |
| *GABRQ* | 6 |
| *HCFC1* | 9 |
| *IDH3G* | 6 |
| *IRAK1* | 9 |
| *L1CAM* | 3 |
| *MAGEA1* | 3 |
| *MECP2* | 26 |
| *NAA10* | 3 |
| *NSDHL* | 1 |
| *OPN1LW* | 3 |
| *OPN1MW* | 3 |
| *OPN1MW2* | 3 |
| *PDZD4* | 7 |
| *PLXNB3* | 6 |
| *PNCK* | 6 |
| *PNMA3* | 4 |
| *PNMA5* | 3 |
| *PNMA6A* | 4 |
| *RENBP* | 3 |
| *SLC6A8* | 12 |
| *TEX28* | 3 |
| *TMEM187* | 3 |
| *ZNF185* | 3 |
| **TOTAL** | **158** |

Scores as given in the AutismKB database (<http://autismkb.cbi.pku.edu.cn/index.php>). The following genes in the interval did not score in the database: *ABCD1*, *BGN*, *CETN2*, *CSAG1*, *CSAG2*, *CSAG3*, *DUSP9*, *FAM58A*, *HAUS7*, *LOC100287428*, *MAGEA12*, *MAGEA2*, *MAGEA2B*, *MAGEA3*, *MAGEA6*, *MIR105-1*, *MIR105-2*, *MIR718*, *MIR767*, *SRPK3*, *SSR4*, *TKTL1*, *TREX2*, *ZFP92*, *ZNF275*.
